# Berufsbezogene Zufriedenheit und Gesundheitswahrnehmung von Tanzpädagoginnen und -pädagogen

**DOI:** 10.1007/s40664-020-00420-8

**Published:** 2021-01-29

**Authors:** Mike Schmidt, Daniela Ohlendorf, Rüdiger Reer, David A. Groneberg, Eileen M. Wanke

**Affiliations:** 1 Fakultät für Bewegungswissenschaft, Institut für Sport und Bewegungsmedizin, Universität Hamburg, Hamburg, Deutschland; 2 Institut für Arbeits‑, Sozial- und Umweltmedizin, Goethe-Universität, Theodor-Stern-Kai 7, Haus 9a, 60590 Frankfurt/Main, Deutschland

**Keywords:** Berufszufriedenheit, Berufliche Gesundheit, Subjektiver Gesundheitszustand, Tanz, Pädagoge/in, Job satisfaction, Occupational health, Subjective health status, Dance, Pedagogue

## Abstract

**Hintergrund:**

Der eigene Körper ist das zentrale Arbeitsinstrument eines*einer Tanzpädagog*in (TP) innerhalb der Bewegungsvermittlung. Bisher fehlen Erkenntnisse über die subjektive Wahrnehmung der eigenen berufsassoziierten Gesundheit und Zufriedenheit sowie die Identifizierung gesundheitsbelastender Berufsmerkmale.

**Methodik:**

Im Rahmen einer fragebogenbasierten Querschnittserhebung wurde eine Kohorte von TP in Deutschland zur eigenen Gesundheit und generellen Berufszufriedenheit und belastenden Aspekten im Zusammenhang mit ihrer Berufsausübung untersucht. Zusätzlich wurden allgemeine anthropometrische und soziodemographische Merkmale erfasst. Neben der Betrachtung der Gesamtkohorte wurde auf geschlechtsspezifische Unterschiede getestet. In die statistische Analyse wurden *n* = 232 TP (m: 51/w: 181) im Alter von 43,1 ± 11,0 Jahren eingeschlossen.

**Ergebnisse:**

Der allgemeine Gesundheitszustand wurde von 85,3 % der Befragten mit „befriedigend“ (26,1 %) bis „sehr gut“ (14,7 %) beurteilt. 59,2 % der Tanzpädagog*innen schätzten ihre Gesundheit „gut“ (35,3 %) bis „sehr gut“ ein. Es herrschte eine hohe Zufriedenheit mit der eigenen Berufsausübung für 80 % der Teilnehmenden. Die TP fühlten sich überwiegend in der Lage (trifft „voll & ganz“ bzw. „eher zu“), mit den physischen (75,7 %) und psychischen Berufsanforderungen (70,3 %) umzugehen. Als belastende Berufsmerkmale in der Eigenwahrnehmung können neben Zukunftsängsten (51,5 %) vor allem arbeitsorganisatorische (fehlende Zeit für Familie und Freunde bei 28,4 %) und ökonomische Aspekte (Einkommensunsicherheit bei 61,0 % und fehlende Altersabsicherung bei 65,7 %) herausgestellt werden.

**Diskussion:**

Die Berufsausübung als TP geht mit einer hohen generellen Zufriedenheit und einem positiven Empfinden des eigenen Gesundheitszustandes einher. Eine Bestätigung dieser positiven Ergebnisse durch Verletzungs- und Erkrankungsstatistiken steht noch aus. Darüber hinaus wäre eine Verbesserung arbeitsorganisatorischer und ökonomischer Aspekte wünschenswert.

## Abstract (en)

**Background:**

The dance teacherʼs own body is considered to be the central working instrument within the movement mediation. Up to now there is a lack of knowledge about the subjective perception of one’s own occupationally associated health and satisfaction as well as the identification of occupational characteristics that are detrimental to health.

**Material and methods:**

Within the framework of a questionnaire-based cross-sectional survey, a cohort of dance teachers in Germany were investigated about dimensions of their own health and general job satisfaction in connection with their profession as a dance teacher. Likert scales were primarily used for these assessments. Additionally, general anthropometric and sociodemographic characteristics were recorded. Beside examining the overall cohort, gender-specific differences were tested. The statistical analysis included *n* = 232 dance teachers (male 51/female 181) aged 43.1 ± 11.0 years.

**Results:**

The general state of health was rated as satisfactory (26.1%) to very good (14.7%) by 85.3% of those surveyed. Of the dance teachers 59.2% even rated their health as good (35.3%) to very good. There was a high degree of satisfaction with their own professional practice for 80% of the participants. Most of the dance teachers felt that they were able to deal with the physical (75.7%) and psychological demands of their profession (70.3%). In addition to fears about the future (51.5%), the main burdensome professional characteristics on dance teachers’ self-perception were organizational aspects of work (lack of time for family and friends in 28.4%) and economic aspects (income insecurity in 61.0% and lack of old age security in 65.7%).

**Conclusion:**

The profession as a dance teacher is accompanied by a high level of general satisfaction and a positive perception of one’s own state of health. A confirmation of these positive results by injury and illness statistics is still pending. In addition, an improvement in work organization and economic aspects would be desirable.

## Hintergrund

Die sport- und bewegungsbezogene Vermittlung technischer, konditioneller, ästhetischer und kompositorischer Inhalte stellt die Hauptarbeit der tanzpädagogischen Tätigkeit dar [8, 16].

Der Aufrechterhaltung der Gesundheit von Tanzpädagog*innen (TP) kann eine Doppelfunktion zugeschrieben werden. Einerseits liegt sie in einem ganz persönlich-individuellen Interesse, um die eigene Lebensführung und existenzsichernde Berufsausübung zu gewährleisten. Andererseits kann ihr eine gesellschaftliche Funktion im Sinne einer erzieherischen, gesundheitsvermittelnden und verantwortungsübernehmenden Vorbildfunktion gegenüber den Schüler*innen zugeordnet werden [11, 19]. Umso erstaunlicher ist es, dass die gesundheitliche Situation solcher Lehrkräfte in der Vergangenheit selten erfasst wurde. Wanke et al. [16] berichteten, dass in ihrer Erhebung 77 % der TP nie an einem Gesundheitsscreening teilgenommen haben. Neben der Erfassung unfallbedingter, akuter Verletzungen, die nach Erkenntnissen von Wanke et al. [17] in dieser Population als sehr gering eingeschätzt werden können, scheinen körperliche und mentale Belastungen eine größere Rolle zu spielen [21].

Als relativ gesichertes Wissen in der arbeitsmedizinischen Betrachtung gilt die Erkenntnis, dass eine allgemeine Berufszufriedenheit in direktem Zusammenhang mit verschiedenen berufsbezogenen Gesundheitsaspekten steht [4], ebenso wie der subjektiv bewertete Gesundheitszustand in Verbindung zum objektiven Gesundheitszustand gebracht werden kann [22]. Korrelationen zwischen Berufszufriedenheit, physischer und mentaler Gesundheit sowie Berufsstress und Fehlzeiten lassen sich ebenso für Lehrer*innen an Schulen zunehmend häufiger nachweisen [2]. Solche Beobachtungen konnten auch innerhalb der speziellen Untergruppe der Sportlehrer*innen festgestellt werden [2, 13]. Eine vergleichbare Betrachtung anderweitig bewegungsvermittelnder Lehrkräfte wie den TP erscheint ebenso sinnvoll, um wertvolle Erkenntnisse über Berufszufriedenheit und den subjektiven Gesundheitszustand zu erhalten. Die Zahl der Berufstätigen in diesem Feld lassen sich allerdings nur schwierig erfassen. Nach Angaben des Deutschen Berufsverbandes für Tanzpädagogik e. V. (DBfT) ist von ca. 900 TP auszugehen, die primär im künstlerischen Tanz tätig sind. Dies umfasst jedoch nur eine Teilgruppe organisierter TP, und es ist aufgrund der ungeschützten Berufsbezeichnung von einer deutlich größeren Population auszugehen. Erste Erkenntnisse aus dieser Berufsgruppe legen nahe, dass sowohl überaus positive als auch negative Aspekte vorliegen. So sehen sich nach Ergebnissen von Wanke et al. [18] 96,6 % der TP in ihrem Traumberuf. Andererseits sind für 85,5 % der Befragten lange krankheitsbedingte Ausfälle nicht vertretbar, und 89,4 % würden sogar unter Schmerzen weiterarbeiten [18]. Hier ist ein deutlicher sozioökonomischer Druck zu vermuten, der ebenfalls einen gesundheitsbelastenden Faktor darstellen könnte [8]. Zusätzlich sehen sich 78,7 % der TP auch nach dem 64. Lebensjahr noch in ihrem Beruf tätig [16]. Internationale Veröffentlichungen und dezidierte Untersuchungen zu diesen selbstwahrgenommenen Gesundheitsdimensionen dieser Population liegen derzeit kaum vor. Basierend auf der Nachfrage bewegungs- und tanzbezogener Angebote kann dieser Thematik jedoch eine nicht unerhebliche Relevanz zugeordnet werden [8]. Nach Angaben des Instituts für Demoskopie (IfD) Allensbach [5] tanzten zwischen 2016 und 2019 ca. 4,1 bzw. 4,3 Mio. Deutsche (ab 14 Jahren) in ihrer Freizeit häufig. Im Falle gelegentlichen Tanzens wird von einer etwa 6‑mal so hohen Partizipation ausgegangen (zwischen 26,7 bzw. 27,5 Mio. Deutsche; [5]).

Das Ziel dieser explorativen, querschnittsbasierten Kohortenstudie war es, Daten über das eigene, berufsbedingte Gesundheitsempfinden, die Berufszufriedenheit sowie Belastungsfaktoren von TP im Rahmen einer anonymen Erhebung (online) zu sammeln. Darüber hinaus sollten mögliche geschlechtsspezifische Unterschiede und Zusammenhänge zwischen einzelnen Belastungsfaktoren herausgestellt werden. Eine Beeinflussung der Ergebnisse durch die COVID-19-Pandemie konnte ausgeschlossen werden, da die Erhebung vor Beginn der Pandemie lag.

## Methodik

### Studiendesign und Studienpopulation

Im Rahmen einer retrospektiven Querschnitts-Kohorten-Studie wurden TP mit einem Online-Fragebogen (in englischer und deutscher Sprache über „survey monkey“) befragt.

Die folgenden Einschlusskriterien wurden vor der Befragung festgelegt:

- Hauptberufliche Ausübung der Tätigkeit als Tanzpädagog*in
- Volljährigkeit (≥ 18 Jahre)
- Angestellt oder freiberuflich
- Überwiegende Arbeit in mindestens einem der folgenden künstlerischen Tanzstile: klassischer Tanz (Ballett), Modern/Jazz Dance oder zeitgenössischer Tanz
- Wohnhaft in Deutschland

Die Studie befolgt wissenschaftsethische Kriterien. Eine Zustimmung zur ethischen Unbedenklichkeit der Studienprozedur erfolgte seitens der Ethikkommission der Charité – Universitätsmedizin Berlin.

Die Studieninformationen sowie freiwillige Einwilligung zur anonymen Teilnahme waren der Befragung vorgeschaltet. Erst mit der aktiven Bestätigung wurde die Onlinebefragung gestartet.

### Fragebogeninhalte

Im Rahmen eines Komplex-Fragebogens wurden u. a. anthropometrische (Alter, Größe, Gewicht), soziodemographische (Bildungs- sowie Berufsabschluss, monatliches Einkommen) sowie in Anlehnung an Wanke et al. [16] tanzspezifische und berufsbezogene (Tanzstile, Berufserfahrung) Merkmale erfragt. Darüber hinaus wurden unter Berücksichtigung und Modifizierung übergeordneter Dimensionen (z. B. „work-privacy conflict“) des COPSOQ [9] psychosoziale Berufsfaktoren, die Berufszufriedenheit sowie der subjektive Gesundheitszustand exploriert. Der subjektive Gesundheitszustand wurde auf einer 6‑stufigen Skala (1 = „sehr gut“ bis 6 = „ungenügend“) eruiert. Zur Einschätzung der allgemeinen Berufszufriedenheit sowie dem Zustimmungsgrad weiterer psychosozialer Berufsfaktoren wurden anstelle 5‑stufiger [9] 7‑stufige Likert-Skalen („Die Antwort stimmt …“: „voll und ganz“, „trifft eher zu“, „etwas“, „teils/teils“, „weniger“, „eher nicht“, „gar nicht“) verwendet.

### Studiendurchführung

Im Rahmen einer Prätestung an einer kleineren Stichprobe von Sportlehrkräften wurden grundlegende Probleme im Umgang mit dem Erhebungsinstrument ausgeschlossen. Die Haupterhebung erfolgte über einen Zeitraum von 3 Monaten. Der Zugang zur anonymen Befragung wurde auf digitalem Wege mit Unterstützung des Deutschen Berufsverbands für Tanzpädagogik e. V. (DBfT e. V.), der Royal Academy of Dance (Germany), der Stiftung TANZ und dem Gemeinnützigen Verein für Tanzmedizin (ta.med e. V.) an potenzielle Kandidaten verschickt.

Um die Rücklaufquote zu erhöhen, wurde mit 3 Erinnerungsmails im gesamten Zeitraum auf die Befragung hingewiesen sowie die Bitte zur Weiterleitung an Kollegen geäußert. Eine finale Rücklaufquote ließ sich aufgrund fehlender Informationen zur Gesamtpopulation der TP in Deutschland nicht berechnen.

### Datenanalyse

Die Datenverarbeitung erfolgte mit Microsoft Excel 2010 (Microsoft Excel (2010), Microsoft Corporation, Redmond, WA, USA). Die deskriptive und analytische Statistik wurde mit IBM SPSS 25 (IBM SPSS Statistics für Windows, Version 25.0, IBM Corp, Armonk, NY, USA) durchgeführt. Allen Tests lag eine zweiseitige Testung und ein Signifikanzniveau von α = 0,05 zugrunde. Die Gruppenunterschiede innerhalb der ordinalskalierten Fragebogenitems, wie den Likert-Skalen, wurden mittels Mann-Whitney-U-Test überprüft. Neben der Angabe der Irrtumswahrscheinlichkeit *p* wurde als Effektstärke r_ES_ mit r_ES_ = z/√*n* angegeben. Zur Identifizierung möglicher Zusammenhänge zwischen den einzelnen Fragebogenitems wurden Spearman-Korrelations-Koeffizienten (r_sp_) berechnet.

## Ergebnisse

### Anthropometrische und soziodemographische Merkmale der Stichprobe

Im gesamten Erhebungszeitraum haben *n* = 241 TP erfolgreich die Umfrage abgeschlossen. Für die finale Datenanalyse wurden die Befragungen von *n* = 232 TP betrachtet, bei denen keine fehlenden Werte für die allgemeinen Personenmerkmale Geschlecht, Alter, Größe und Gewicht zu verzeichnen waren.

Tab. 1 gibt eine Übersicht über wichtige anthropometrische Merkmale der Stichprobe. Mit einem Anteil von 78 % (*n* = 181) überwogen die weiblichen TP deutlich ihre männlichen Kollegen (22 % bzw. *n* = 51).

**Table 1:** 

Merkmal	*n*	MW	SD	95 % KI
*Alter (Jahre)*				
Gesamt	232	43,1	11,0	41,7–44,5
Frauen	181	42,0	10,5	40,5–43,5
Männer	51	46,9	11,9	43,6–50,3
*Größe (cm)*				
Gesamt	232	170,0	7,7	169,0–171,0
Frauen	181	167,8	6,2	166,9–168,7
Männer	51	177,8	7,6	175,7–179,9
*Gewicht (kg)*				
Gesamt	232	61,7	10,8	60,3–63,1
Frauen	181	58,3	8,2	57,1–59,5
Männer	51	74,0	10,1	71,1–76,8
BMI (kg*m^*−2*^*)*				
Gesamt	232	21,3	2,7	20,9–21,6
Frauen	181	20,7	2,4	20,3–21,0
Männer	51	23,4	2,6	22,6–24,1

**Figure 1:**
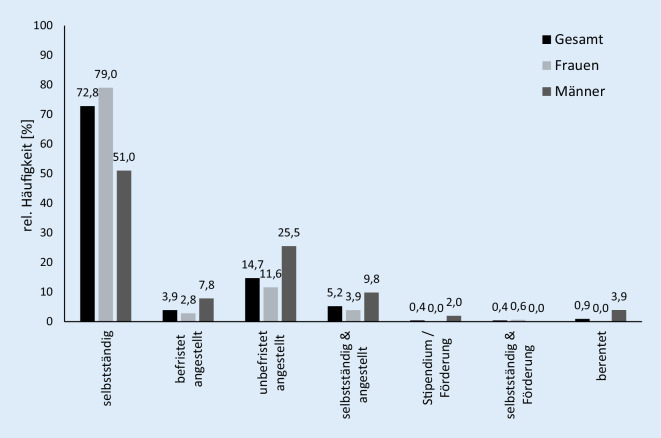

Die TP waren zumeist selbstständig tätig (72,8 %; Abb. 1) und der Großteil (90,1 %) besaß ein monatliches Nettoeinkommen ≤ 3500 €. Etwa drei Viertel der Rückmeldungen (78,7 %) lag bei weniger als 2500 € und etwa ein Drittel (31,2 %) bei weniger als 1000 € pro Monat. Es bestand ein signifikanter Unterschied im monatlichen Verdienst zwischen weiblichen und männlichen Lehrkräften (r_ES_ = 0,22; *p* = 0,008). Lediglich 24,7 % der Lehrkräfte gaben an, über keine Hochschulreife zu verfügen und 8,5 % absolvierten sogar ein Hochschulstudium.

## Subjektive Beurteilung des allgemeinen Gesundheitszustandes

**Figure 2:**
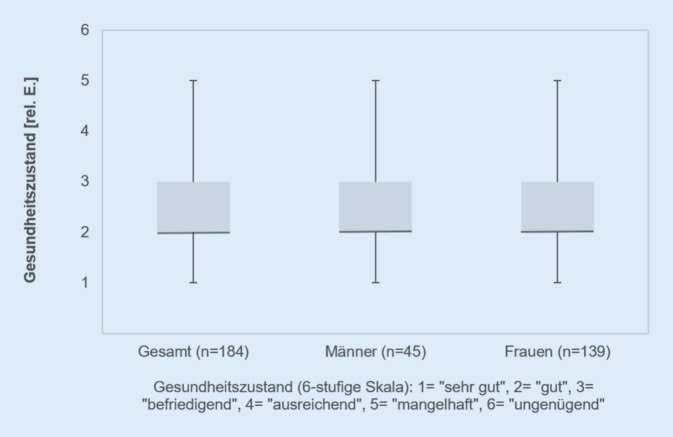

Der eigene Gesundheitszustand wurde von 85,3 % mit „befriedigend“ (26,1 %), „gut“ (44,5 %) oder „sehr gut“ (14,7 %) bewertet. Lediglich 14,7 % beurteilten ihren Gesundheitszustand mit „ausreichend“ (8,2 %) oder gar „mangelhaft“ (6,5 %). Die Einschätzung „ungenügend“ wurde von niemandem ausgewählt. Die geschlechtsspezifischen Unterschiede waren nicht signifikant (Abb. 2).

## Allgemeine Berufszufriedenheit und -umstände

Es konnte eine insgesamt sehr hohe allgemeine Zufriedenheit mit der Berufsausübung als TP gezeigt werden. 34,4 % der TP waren „voll und ganz“ zufrieden mit ihrem Beruf, gefolgt von 45,6 %, die die zweithöchste Zufriedenheitseinschätzung „trifft eher zu“ angaben. Lediglich 12,6 % der Befragten beurteilten ihren Zufriedenheitsgrad mit „teils/teils“ oder schlechter.

**Figure 3:**
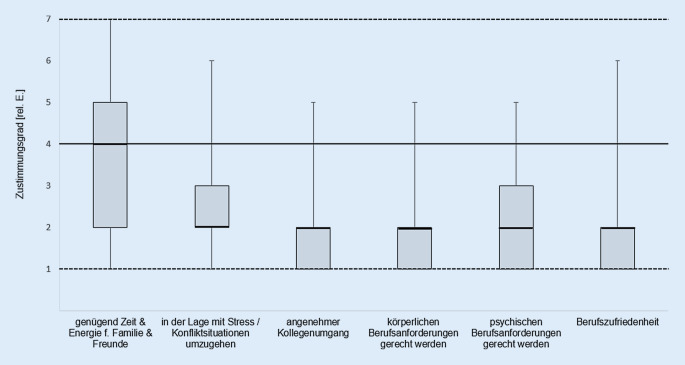

Ähnliches ließ sich für den Kontakt zu den Kollegen abbilden. Mit „voll und ganz“ bzw. „trifft eher zu“ stimmten 45,0 % bzw. 40,3 % der TP einem angenehmen Kollegenkontakt zu. Darüber hinaus sahen sich die Lehrkräfte in der Lage, den täglichen, körperlichen Belastungen gerecht zu werden. Etwas geringer fiel die Zustimmung zum Umgang mit den psychischen Berufsanforderungen sowie Stress- und Konfliktsituationen aus. Die größte Aussageheterogenität konnte für die Frage nach ausreichend Zeit und Energie für Familie und Freunde verzeichnet werden. Diese wurde von 28,4 % tendenziell negiert („weniger“ bis „gar nicht“) bzw. von 51,7 % aller mit „teils/teils“ oder schlechter bewertet (Abb. 3).

Es zeigten sich geschlechtsspezifische Unterschiede im Umgang mit Stress- und Konfliktsituationen (r_ES_ = 0,19; *p* = 0,003) sowie dem Empfinden den psychischen Berufsanforderungen gerecht zu werden (r_ES_ = 0,14; *p* = 0,039). Die weiblichen TP sahen sich in beiden Merkmalen etwas stärker belastet als ihre Kollegen.

## Beurteilung weiterer Berufsaspekte als Belastungsfaktoren

**Figure 4:**
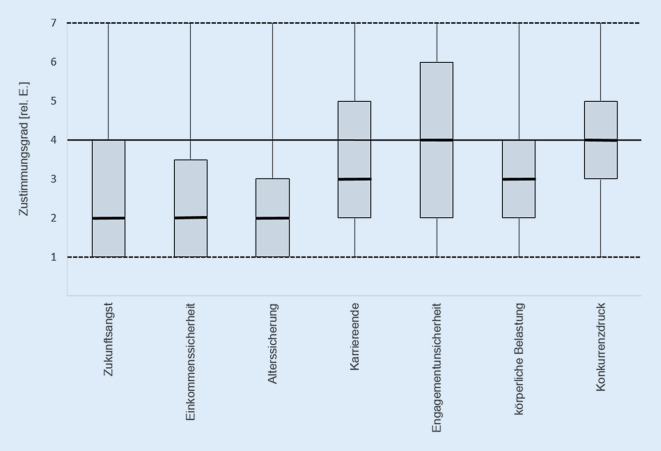

Abb. 4 gibt einen Überblick über Berufsaspekte und das mit diesen jeweils assoziierte Belastungsempfinden der TP. Es wurde deutlich, dass mehr als die Hälfte (51,5 %) Zukunftsängste äußerten und 61,0 % der Befragten einen belastenden Faktor in der Absicherung ihres Einkommens sahen. Weiterhin belastete 65,7 % aller TP der Aspekt der Altersabsicherung. Hier zeigte sich ein signifikanter geschlechtsspezifischer Unterschied. So ging von männlichen TP ein höherer Besorgnisgrad aus als von den weiblichen TP (r_ES_ = 0,17; *p* = 0,012).

Die Fragen nach einem vorzeitigen Karriereende, Unsicherheiten in einem konkreten Engagement, möglichem Konkurrenzdruck sowie der körperlichen Belastung im Berufszusammenhang wurden mit geringerer Besorgnis verzeichnet. In diesen Merkmalen konnten keine geschlechtsspezifischen Unterschiede registriert werden.

## Zusammenhänge zwischen Gesundheitszustand, Zufriedenheit und Einkommen

Es konnte festgestellt werden, dass zwischen der Bewertung des eigenen Gesundheitszustandes und den dargestellten Berufsmerkmalen (Abb. 3 und 4) lediglich kleine oder keine Zusammenhänge (alle Korrelationskoeffizienten < 0,5) gefunden werden konnten. Der höchste Zusammenhang zum Gesundheitszustand zeigte sich in Verbindung mit der Beurteilung der körperlichen Belastung (r_SP_ = −0,43; *p* < 0,000). Die Korrelationen des Gesundheitszustandes zur generellen Berufszufriedenheit (r_SP_ = 0,18; *p* = 0,019) und dem Einkommen (r_SP_ = −0,19; *p* = 0,029) fielen äußerst klein aus. In direkterer Beziehung standen die Berufszufriedenheit und das Einkommen, jedoch ebenfalls nur mit einem kleinen linearen Zusammenhang (r_SP_ = −0,37; *p* < 0,000).

## Berufsmerkmale in der Belastungsbeurteilung mit direktem Zusammenhang

Tab. 2 gibt eine Übersicht über alle mittleren und hohen Zusammenhänge zwischen den einzelnen Berufsmerkmalen, die in den Abb. 3 und 4dargestellt sind. Insgesamt konnten zwei hohe und acht mittlere positive Zusammenhänge ermittelt werden. Die beiden hohen Korrelationen bestanden jeweils zwischen dem Merkmal „Einkommenssicherheit“ und den Merkmalen „Zukunftsangst“ (r_SP_ = 0,803; *p* < 0,000) bzw. „Altersabsicherung“ (r_SP_ = 0,741; *p* < 0,000). Daraus kann geschlossen werden, dass Personen, die sich von ihrer Einkommenssituation belastet fühlten, ebenfalls in ihrer Altersabsicherung sowie Zukunftssicherung Besorgnis erregende Berufsaspekte sahen.

**Table 2:** 

Merkmalspaarung			r_SP_	*p*
Einkommenssicherheit	–	Zukunftsangst	0,803	< 0,000
Einkommenssicherheit	–	Altersabsicherung	0,741	< 0,000
Zukunftsangst	–	Altersabsicherung	0,665	< 0,000
Psychische Anforderungen	–	Physische Anforderungen	0,563	< 0,000
Altersabsicherung	–	Vorzeitiges Karriereende	0,549	< 0,000
Zukunftsangst	–	Unsicherheit in Engagement	0,539	< 0,000
Einkommenssicherheit	–	Unsicherheit in Engagement	0,534	< 0,000
Vorzeitiges Karriereende	–	Unsicherheit in Engagement	0,525	< 0,000
Psychische Anforderungen	–	Umgang mit Stress- & Konfliktsituationen	0,508	< 0,000
Zukunftsangst	–	Vorzeitiges Karriereende	0,507	< 0,000

Merkmale zur Einschätzung der physischen und psychischen Anforderungen in Verbindung zu Merkmalen mit berufswirtschaftlichem und -organisatorischem Hintergrund zeigten maximal kleine Zusammenhänge. Hierbei wurde der größte Zusammenhang (r_SP_ = 0,470; *p* < 0,000) zwischen der körperlichen Belastung und der Angst vor einem vorzeitigen Karriereende konstatiert. Weiterhin bestand zwischen der Beurteilung physischer Anforderungen und den körperlichen Belastungen nur ein sehr geringer Zusammenhang (r_SP_ = −0,292; *p* < 0,000).

## Diskussion

### Subjektiver Gesundheitszustand

Der subjektiv wahrgenommene Gesundheitszustand kann für die gesamte Stichprobe als gut beurteilt werden, wobei keine geschlechtsbezogenen Unterschiede bestehen. Der Personenanteil (59,2 %) mit einer guten oder sehr guten Beurteilung entspricht etwa den Beobachtungen in der deutschen Gesamtbevölkerung innerhalb gleicher Altersstrukturen [10]. So bewerten ca. 55 % der 40- bis 49-jährigen Männer und Frauen ihren subjektiven Gesundheitszustand mit gut bis sehr gut [10]. Es kann vermutet werden, dass für diesen Großteil ebenfalls ein guter objektiver Gesundheitszustand vorliegt [22], auch wenn festzuhalten ist, dass selbstbewertete und objektive Gesundheit nicht gleichzusetzen sind [10, 22], sondern vielmehr als Indikator zu betrachten wären. Interessant ist, dass laut Robert Koch-Institut [10] in der deutschen Referenzpopulation lediglich etwa 2 % eine schlechte Bewertung ihrer Gesundheit vorgenommen haben. Der Anteil innerhalb der TP mit 6,5 % (Bewertung: „mangelhaft“) fällt deutlich höher (≈ 3-fach) aus. Es ist zu erwarten, dass für diese Untergruppe auch objektive Gesundheitseinschätzungen (z. B. Krankheitsprävalenzen) schlechter ausfallen [22]. Aus den Beobachtungen muss vermutet werden, dass positive Gesundheitseffekte durch die Berufsausübung gering ausfallen und in gleichem Maße nicht von bedeutenden Verzerrungen im Kontext von Healthier-Worker-Effekten [6] auszugehen ist, bedürfen jedoch weiterer objektiver Gesundheitseinschätzungen.

## Generelle Berufszufriedenheit

Bezogen auf die generelle Berufszufriedenheit konnte ein sehr hoher Zufriedenheitsgrad eruiert werden. Dies untermauert die Feststellung von Wanke et al. [18], dass es sich für die große Mehrheit um die Ausübung des Traumberufes handelt und stellt zusätzlich einen günstigen Umstand für eine gesundheitserhaltende Berufsausübung dar. Eine mögliche Erklärung für dieses Phänomen könnte sein, dass sich ein Großteil der Population in einer selbstständigen Arbeitssituation wiederfindet und somit über die direkten Arbeitsinhalte und die einhergehenden arbeitsstrukturellen Bedingungen entscheiden kann. So zeigten Skaalvik und Skaalvik [14] an 2569 Lehrer*innen den direkten Zusammenhang zwischen Arbeitszufriedenheit und arbeitsbezogener Autonomie (r = 0,37). Im Umkehrschluss kann ebenso vermutet werden, dass durch den hohen Zufriedenheitsgrad ein sehr geringes Risiko für ein berufsbedingtes Burnout besteht.

## Berufsbelastung

TP sehen sich in der Lage, mit bestehenden körperlichen und psychischen Anforderungen umzugehen und diesen gerecht zu werden. Das kollegiale Berufsumfeld wird als angenehm empfunden. Es lässt sich vermuten, dass TP in einer Vielzahl ihrer direkten Berufsaufgaben, wie der sportmotorischen, choreographischen und ästhetischen Vermittlung, ein hohes Gefühl von Kontrolle und Kohärenz zu den eigenen Berufsanforderungen sehen und diese Bestandteile als gesundheitsstärkende Ressource wirken könnten. Die geschlechtsspezifischen Unterschiede zu Gunsten der männlichen TP mit einem geringeren Stress- und Konfliktempfinden sowie einem höheren Empfinden, psychischen Berufsanforderungen gerecht zu werden, ließen sich zeigen. Diese allgemeine Tendenz zeichnet sich auch in der Literatur ab [1, 6]. Von einer praktischen Relevanz kann jedoch aufgrund der kleinen Effekte nicht ausgegangen werden. Zusätzlich können diese Geschlechtsunterschiede nach Alters- oder Bildungsgradadjustierung unter Umständen verschwinden [7]. Weiterhin zeigen die Ergebnisse, dass die Berufsausübung mit zeitlichen und energetischen Engpässen für das soziale Umfeld (Familie/Freunde) verbunden sein kann. Es lässt sich jedoch keine einheitliche Situation innerhalb der Stichprobe feststellen. Auf welche Ursache dieser Umstand zurückzuführen ist, bleibt spekulativ. Auffällig ist, dass TP vor allem besorgt sind über ihre Zukunfts‑, Einkommens- sowie Alterssicherung. Diese Merkmale belasten die TP deutlich. Schaut man sich den hohen Anteil selbstständig Berufstätiger und das zumeist niedrige Einkommen an, erscheint dies nachvollziehbar und deckt sich mit früheren Erkenntnissen [18]. Zusätzlich ist zu vermuten, dass die COVID-19-bedingten Einschränkungen zu einer Verschlechterung der Berufssituation und sozioökonomischen Lage führen könnten.

## Körperliche Arbeit mit ambivalenter Wirkung

Von der eigenen Körperarbeit scheinen sowohl positive als auch belastende Wirkungen auszugehen. So sehen sich die TP einerseits in der Lage, den physischen Anforderungen gerecht zu werden, andererseits wird die körperliche Arbeit als Belastungsfaktor wahrgenommen. Diese ambivalente Wirkung der physischen Aktivität im Beruf als TP lässt sich ebenfalls aus den Arbeiten von Dahlström [3], Schmidt et al. [12] und Wanke et al. [20] ableiten. So belegen Dahlström [3] und Wanke et al. [20], dass körperliche Beanspruchungen des Herz-Kreislauf-Systems im Unterrichtsgeschehen in ihren Höchstbelastungen durchaus submaximale bis maximale Intensitäten erreichen können. Andererseits fallen durchschnittliche Herz-Kreislauf-Belastungen im Unterrichtsverlauf häufig gering bis moderat aus [20], und mit der Berufsausübung kann nicht automatisch eine höhere Fitness verbunden werden [12]. Eine ähnliche Beobachtung im Hinblick auf die Wirkung berufsbezogener körperlicher Aktivität lässt sich in der Studie von Bogaert et al. [2] für Sportlehrer*innen finden. Einerseits zeigten sie im Vergleich zu ihren restlichen Lehrerkolleg*innen eine signifikant bessere mentale Gesundheit sowie geringere Stresslevel und eine Begründung über eine höhere physische Aktivität im Zuge ihrer Berufsausübung erscheint naheliegend. Demgegenüber stellen die Autoren jedoch fest, dass in der Gesamtstichprobe aller Lehrkräfte ein höherer Umfang physischer Berufsaktivität teilweise mit geringeren Gesundheitsoutcomes assoziiert war [2]. Sie erklären ihre Ergebnisse mit dem spezifischen Aufgabencharakter der im Rahmen der Lehrtätigkeit ausgeführten physischen Aktivitäten [2]. Solche Unterschiede in der Arbeit mit dem eigenen Körper und eine hohe Individualität im Unterrichtsprozess lassen sich ebenso für die TP vermuten. So bringt beispielsweise der Einfluss der zu unterrichtenden Zielgruppe einen deutlichen Belastungsunterschied mit sich [21]. Festzuhalten ist, dass die größte Assoziation mit einem vorzeitigen Karriereaus zur körperlichen Belastung bestand und somit dem Umgang und der differenzierten Betrachtung dieser berufsbezogenen Körperarbeit eine Schlüsselrolle zugeordnet werden muss.

## Limitationen

Da es sich um eine Fragebogenerhebung handelte, konnte nicht ausgeschlossen werden, dass die abgebildeten Belastungseinschätzungen die tatsächlichen Zustände der untersuchten Population leicht über- oder unterschätzen. Teilweise verweigerte Antworten könnten zu solchen Verzerrungen führen. So haben beispielsweise 20,7 % der Stichprobe keine Bewertung zum eigenen allgemeinen Gesundheitszustand abgegeben. Ob dadurch eine Überschätzung und damit positivere Sicht auf die Stichprobe zustande kam, kann letztlich nicht beantwortet werden. Ähnliches gilt für Fragebogenitems zu sensiblen Personenmerkmalen wie beispielsweise dem monatlichen Verdienst. Trotz Anonymität wurde dieses Item von 41,5 % nicht beantwortet. Von einer bewussten Verfälschung bestehender Antworten gehen die Autoren jedoch aufgrund des anonymen und freiwilligen Befragungscharakters nicht aus. Zudem erfolgte die Akquise vordergründig über Verbandsstrukturen, mit denen von einer gewissen Vertraulichkeit auszugehen war. Die bereits in früheren Arbeiten [8, 21] und erneut beschriebenen Ängste vor finanzieller Unsicherheit lassen vermuten, dass vor allem geringere Einkommen ohne Angaben blieben und somit eine Überschätzung der Einkommenssituation nicht ausgeschlossen werden kann.

Weiterhin soll angemerkt werden, dass eine direkte Vergleichbarkeit zwischen den Ergebnissen des Robert Koch Institutes (RKI) [10] zum subjektiven Gesundheitszustand und den Ergebnissen dieser Arbeit aufgrund leicht unterschiedlicher Erhebungsinstrumenten (5- vs. 6‑fach abgestufte Ratingskala) nicht gegeben ist. Vielmehr soll es sich um eine grobe Einordnung der Befunde zur entsprechenden (deutschen) Gesamtbevölkerung handeln. Die Autoren möchten den explorativen Charakter der Studie explizit hervorheben und betonen, dass ihnen bewusst ist, dass aufgrund multipler Testungen ein zufälliges Auftreten einzelner Testergebnisse nicht ausgeschlossen werden kann und herausgearbeitete Hypothesen erneuter Absicherung in Folgearbeiten bedürfen [15]. Im Rahmen dieser Arbeit wurde deshalb auf eine *p*-Wert-Adjustierung verzichtet.

Trotz der beschriebenen Grenzen liefert die vorliegende Arbeit wertvolle Erkenntnisse zur Bewertung der subjektiven Gesundheit, dem Belastungsempfinden und der Zufriedenheit in einer Population, in der Betriebsärzte, Arbeitssicherheit und Abteilungen für Betriebliche Gesundheitsförderung praktisch nicht existent sind.

## Ausblick

Nach subjektiver Einschätzung empfinden TP eine hohe allgemeine Berufszufriedenheit und stehen ihren körperlichen und mentalen Anforderungen sowie ihrem Gesundheitszustand positive gegenüber. Lediglich sozioökonomische sowie psychosoziale Aspekte nehmen eine gesundheitsbelastende Rolle ein. Um aus der subjektiven Belastungssituation Aussagen über die Beanspruchung von TP geben zu können, bedarf es in Folgearbeiten einer Relativierung und Kontextualisierung der subjektiven Einschätzungen anhand objektiver Gesundheits- sowie Krankheits- und Verletzungsmerkmale. In diesem Kontext sollten Fragen zu möglichen Healthy-Worker-Effekten berücksichtigt werden. Zusätzlich sollten die Auswirkungen der COVID-19-Pandemie auf die Berufssituation untersucht werden, da gravierende Veränderungen sämtlicher Berufsmerkmale im engeren (Unterrichtsdurchführung) sowie im weiteren Sinne (sozioökonomische Aspekte, Arbeitszufriedenheit etc.) zu erwarten sind.

## Fazit für die Praxis

- Als psychische Stressoren können primär ökonomische und arbeitsorganisatorische Faktoren identifiziert werden.
- Es kann vermutet werden, dass von der eigenen Körperarbeit sowohl eine gesundheitsförderliche als auch -hinderliche Wirkung ausgehen kann. Eine differenziertere Betrachtung der speziellen Körperarbeit im konkreten Arbeitskontext erscheint zwingend erforderlich.
- Eine regelmäßige und bewusste Reflexion berufsbezogener Zufriedenheit und Belastungseinschätzungen könnte eine einfache und ressourcengünstige Methode zur Einschätzung des eigenen subjektiven Gesundheitszustandes darstellen, die eigenständig von den TP im Rahmen eines primärpräventiven Gedankens empfohlen werden kann.
